# Characteristics of Shiga Toxin-Producing *Escherichia coli* Circulating in Asymptomatic Food Handlers

**DOI:** 10.3390/toxins15110640

**Published:** 2023-11-02

**Authors:** Xinxia Sui, Xi Yang, Ming Luo, Hua Wang, Qian Liu, Hui Sun, Yujuan Jin, Yannong Wu, Xiangning Bai, Yanwen Xiong

**Affiliations:** 1 National Key Laboratory of Intelligent Tracking and Forecasting for Infectious Diseases, National Institute for Communicable Disease Control and Prevention, Chinese Center for Disease Control and Prevention, Beijing 102206, China; 2 Yulin Center for Disease Control and Prevention, Yulin 537000, China; 3 Longgang Center for Disease Control and Prevention, Shenzhen 518172, China; 4 Division of Laboratory Medicine, Oslo University Hospital, 0372 Oslo, Norway

**Keywords:** *Escherichia coli*, Shiga toxin, food handlers, Stx prophage, hemolytic uremic syndrome

## Abstract

Shiga toxin-producing *Escherichia coli* (STEC) is a foodborne zoonotic pathogen that causes diarrhea, hemorrhagic colitis (HC), and hemolytic uremic syndrome (HUS) worldwide. Since the infection can be asymptomatic, the circulation of STEC in some asymptomatic carriers, especially in healthy-food-related professionals, is not yet well understood. In this study, a total of 3987 anal swab samples from asymptomatic food handlers were collected, and ten swabs recovered STEC strains (0.251%). Of the ten STEC isolates, seven serotypes and eight sequence types (ST) were determined using whole genome sequencing (WGS). Two *stx1* subtypes (*stx1a* and *stx1c*) and four *stx2* subtypes (*stx2a*, *stx2b*, *stx2d*, and *stx2e*) were detected. Seven different insertion sites were found in fourteen Stx prophages, and the *dmsB* and *yfhL* were the newly identified insertion sites. The ten strains showed the variable Stx transcription levels after the mitomycin C induction. The whole-genome phylogeny indicated that the strains from the asymptomatic food handlers were genetically distant from the strains of HUS patients. The STEC isolates circulating in asymptomatic carriers might pose a low potential to cause disease.

## 1. Introduction

Shiga toxin-producing *Escherichia coli* (STEC) represents a well-known group of foodborne zoonotic pathogens, causing sporadic cases and outbreaks of diarrhea, hemorrhagic colitis (HC), and hemolytic uremic syndrome (HUS) worldwide [1]. STEC has emerged as a great public health concern due to the significant risk of serious and potentially life-threatening complications of infection, e.g., HUS. It was estimated that on average, there are 2.8 million acute infections globally each year, with a greater rate of infection in children [2].

Shiga toxin (Stx) is the major virulence factor of STEC. Stx consists of two immunologically distinct types: Stx1 and Stx2, which can be further divided into several subtypes based on variations in amino acid sequences. Stx1 includes three subtypes (Stx1a, Stx1c, and Stx1d), while Stx2 contains at least seven subtypes (Stx2a to Stx2g) [3]. Stx1/Stx2 subtypes exhibit significant differences in biological activity, including serologic reactivity, receptor binding, and toxin potency. Stx2 production is more often associated with severe diseases than strains producing Stx1 [4]. In recent years, several novel Stx2 subtypes have been identified, including Stx2h to Stx2m, and Stx2o [5,6,7,8,9].

Stx is encoded in the late region of the lambdoid bacteriophage named Stx prophage. The induction of Stx prophages can significantly increase Stx production and trigger phage-mediated lysis, thereby causing Stx release [10]. Stx prophages are mobile elements that can mediate the horizontal gene transfer and, therefore, contribute to the dissemination of Stx genes, leading to the emergence of new STEC strains or hybrid pathotypes [11,12,13]. Thus, Stx-converting prophage is recognized as a key driver for STEC pathogenesis [14].

Ruminants are considered the primary STEC reservoir [15], especially cattle [16]. STEC has also been detected in other domesticated animals, such as pigs [17]. Human infection can occur through the ingestion of contaminated foods, person-to-person transmission, or contact with animal reservoirs [18,19,20]. The STEC infectious dose is much lower than that of many other enteropathogens [21]. They have an estimated 10~100 organisms capable of causing infection, which increases the risk of infection from exposure and facilitates person-to-person transmission [22].

Previous studies have reported on STEC isolated from various animals, foods, and diarrheal patients [23,24]. Since the infection can be asymptomatic, the circulation of STEC in asymptomatic carriers, especially in healthy-food-related professionals, is not yet well understood. Herein, we conducted a study to investigate the prevalence and characteristics of STEC isolated from food handlers in China to evaluate the potential public health risk of infection.

## 2. Results

### 2.1. Occurrence of STEC in Asymptomatic Food Handlers

The samples were collected from three provinces in China, including Guangxi, Guangdong, and Qinghai. In total, 10 STEC strains (0.251%) were isolated from 10 out of 3987 anal swab samples from asymptomatic food handlers (Table 1). The different STEC culture-positive rates were observed in Guangdong (0.222%), Qinghai (2.041%), and Guangxi (0.232%), respectively.

### 2.2. Molecular Characteristics of Asymptomatic Food Handlers-Derived STEC Isolates

Serotyping and MLST revealed that ten STEC strains were genetically diverse. Seven rare serotypes (i.e., O43:H2, O91:H14, O8:H19, O178:H7, O122ab:H8, O21:H25, and O122ac:H19) and eight sequence types (ST) (i.e., ST937, ST33, ST40, ST21, ST278, ST971, ST2038, and ST75) were identified. Four strains harbored *stx1* only, two strains contained *stx2* only, and four strains possessed both *stx1* and *stx2*. Two *stx1* subtypes (*stx1a* and *stx1c*) and four *stx2* subtypes (*stx2a*, *stx2b*, *stx2d*, and *stx2e*) were detected among these STEC strains (Table 2).

### 2.3. Genetic Features of Stx Prophages

**Figure 1:**
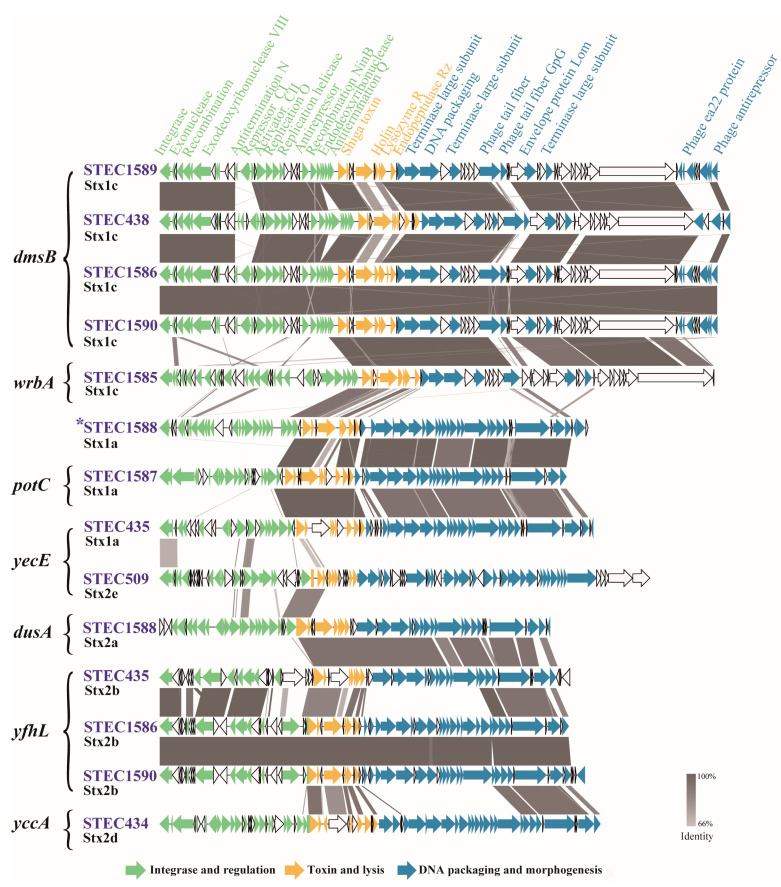
Architecture of fourteen prophages from ten STEC isolates. Easyfig plot was used to compare the fourteen prophages. Arrows indicate gene directions. Phage integrase and regulation genes are shown in green; toxin and lysis genes are shown in yellow; DNA packaging and morphogenesis genes are shown in blue; and the genes encoding hypothetic proteins are shown in white. * The insertion site was undetermined.

Fourteen complete sequences of prophages were predicted and extracted from 10 complete STEC genome sequences. The 14 Stx prophages were characterized in terms of insertion site, genetic content, and functional structure (Figure 1). Their sizes ranged from 45,120 bp to 64,251 bp, with predicted CDSs ranging from 61 to 89 (Table S1). Seven different insertion sites were found. Four prophages were integrated into the coding sequence of *dmsB* (dimethyl sulfoxide reductase subunit B); three were integrated into the *yfhL* (putative 4Fe-4S cluster-containing protein YfhL); and two were inserted into *yecE* (DUF72 domain-containing protein YecE). Four prophages were integrated into *yccA* (Modulator of FtsH protease YccA), *wrbA* (NAD(P)H: quinone oxidoreductase), *potC* (spermidine preferential ABC transporter membrane subunit PotC), or *dusA* (encoding tRNA dihydroxyuridine synthase A), respectively. The insertion site of Stx1a prophage in strain STEC1588 was undetermined (Figure 1).

### 2.4. Variable Stx mRNA Expression Levels

**Figure 2:**
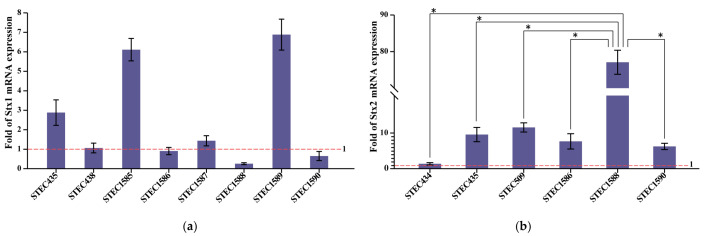
Fold of Stx mRNA transcription in induced state relative to non-induced state. Non-induced state was used as calibrator and data are means ± standard deviations from three independent replicates. Fold change greater than one indicates mRNA transcription increased. (**a**) Fold of Stx1 mRNA transcription; (**b**) Fold of Stx2 mRNA transcription. * It indicates statistically significant differences.

To evaluate the Stx expression at the transcription level, all ten of the STEC strains were induced using mitomycin C. Among the eight Stx1-producing strains, four of them (STEC435, STEC1585, STEC1587, and STEC1589) showed a higher level of Stx1 mRNA transcription after induction (Figure 2a). The Stx2 mRNA transcription of the six Stx2-producing strains increased after induction (Figure 2b). Notably, strain STEC1588, producing Stx2a, showed higher inducibility than the other Stx2 subtype strains (*p*-value ≤ 0.05).

### 2.5. Antimicrobial Resistance (AMR) and AMR Genes

Among the ten STEC isolates, strain STEC438 showed resistance to tetracycline and streptomycin, and strain STEC509 showed resistance to tetracycline. The predicted resistance genotype matched well with the phenotypic resistance profiles. A new fosfomycin-resistant gene, *fosA7*, was identified in three STEC strains (STEC1586, STEC1589, and STEC1590), but the fosfomycin-resistant phenotypes were not tested in this study (Table 2).

### 2.6. Phylogenetic Relationships of STEC Strains from This Study and Other Sources

**Figure 3:**
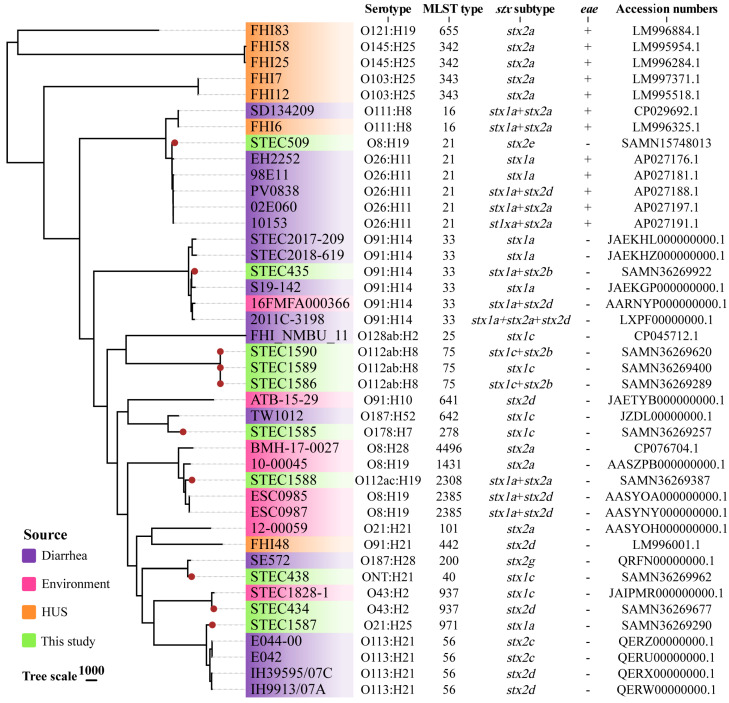
Phylogenetic tree based on core-genome single-nucleotide polymorphisms (SNPs) using the maximum likelihood method. The source, serotype, MLST type, *stx* subtype, *eae*, and accession numbers of all strains are shown. The red circles represent the strains isolated from this study.

To investigate the phylogenetic relationship of the STEC strains between asymptomatic food handlers and reference strains, a wgSNP-based phylogeny tree was constructed. The reference genomes included the “Big 6” non-O157 serogroups (O26, O45, O103, O111, O121, and O145) strains and strains that hold identical serogroups in this study. The analysis showed that the 10 STEC strains from asymptomatic food handlers in this study were generally dispersed; the three strains (STEC1586, STEC1589, and STEC1590) that were isolated from the same location and shared the same serotype and ST type were clustered together with less than 10 SNPs. The other seven strains in this study were distributed separately; among these, STEC1587 and STEC509 clustered with the “Big 6” diarrhea patient-derived strains with 564 and 329 SNPs, respectively. The strain STEC438 clustered with a diarrhea patient-derived strain with 384 SNPs. Two strains (STEC434 and STEC1588) clustered with environment-derived strains, and the SNP distances ranged from 300 to 600. All asymptomatic food handlers-derived STEC strains were separated from HUS strains (Figure 3).

## 3. Discussion

Many outbreaks and sporadic infections caused by STEC O157:H7/NM have been reported worldwide [25,26,27]. Of note, severe infections caused by non-O157 STEC strains have been increasingly reported in recent years. For example, a large outbreak caused by STEC O104:H4 infected 3816 people and caused 54 deaths in Germany in 2011, affecting other European countries and North America [28]. The incidence of non-O157 STEC infections has risen from 0.19 per 100,000 in 2007 to 0.79 per 100,000 in 2014 in the USA. Non-O157 STEC strains were more common causes of acute diarrhea than O157 strains [29]. In China, an O157:H7 outbreak was reported in Xuzhou in 1999, leading to the hospitalization of 195 HUS patients and 177 deaths [30]. Subsequently, 20-year national surveillance (2001 to 2021) revealed that the isolation rate of STEC O157:H7 in diarrhea patients was 0.07% in Xuzhou City, Jiangsu Province, China [31]. Recently, a foodborne outbreak of diarrhea caused by non-O157 STEC was reported for the first time in China. The STEC O146:H10 strains were isolated from a diarrheal patient and a kitchen worker, respectively [32].

After food safety measures and STEC surveillance system were established in some countries, STEC infection became a notifiable disease. Asymptomatic STEC carriers are legally restricted from working as food handlers. To prevent the spread of infection via food, food handlers are required to carry out routine fecal examinations for a wide variety of infectious pathogens, including STEC [33,34]. The prevalence of STEC in asymptomatic infants and adults has been investigated in several areas. A study in France described STEC carriage in a large cohort, where the estimated rate was 1% among 959 healthy French infants [35]. A cross-sectional analysis in Germany showed a STEC prevalence of 0.5% among 224 asymptomatic children [36]. In Japan, a study showed that 398 out of 472,734 (0.08%) healthy adults were positive for STEC [37]. Since STEC infection is not a mandatory notifiable disease in China, the prevalence of STEC in the population is unclear. In this study, 0.251% of 3987 detected samples from asymptomatic food handlers were positive for STEC. The detection rate for STEC strains was highest in Qinghai (2.041%) compared to Guangdong (0.222%) and Guangxi (0.232%); however, the sample number for Qinghai was relatively small, which warranted further investigations using greater numbers in this region. The prevalence was similar in Guangdong and Guangxi, although the sampling spanned several years. The comparison among different regions should be explained with caution because the prevalence differed in terms of the demography, as well as sampling season, residential areas, and detection methods. It is worth noting that some PCR-positive samples were negative by culture in the study. Several factors could contribute to the failure to isolate STEC from the *stx*-positive samples, for example, the perturbation of background microflora, the low levels of *stx* in the samples, and the interference of free Stx phages [38]. The use of different STEC-selective culture media and isolation methods can also have an impact on STEC strain isolation [39]. The O:H serotyping of STEC strains has been used widely to evaluate the potential to cause severe diseases. Even though hundreds of different serotypes have been characterized and new ones continue to be found, the serogroups O26, O45, O103, O111, O121, and O145 (also referred to as the “top six”) have been suggested to be the most related to severe human diseases [40]. A high diversity of serotypes was recognized among the ten strains in our study. The predominant serotype in food handler-derived strains was O112:H8, different from that of diarrhea patient-derived strains in China [41]. The results also differ from a Japanese study, where O26:H11 was the predominant serotype from asymptomatic food handlers-isolated strains [34]. The O112 serogroup has been isolated from beef and caprine samples but not from human samples [42,43]. The serotype O8:H19 in this study had been reported as the most frequent serotype isolated from dairy farm environments [44,45]. The “top 6” non-O157 STEC serogroups were not identified in this study. This implies that asymptomatic carriers are not the reservoirs of predominant serotypes.

Stx2 was reported to be more frequently associated with severe clinical diseases in humans than Stx1 [46]. Stx1a has been linked to human illness; however, STEC that produce subtypes Stx2a, Stx2c, and Stx2d are more often correlated with the development of HC and HUS [47,48]. STEC carrying Stx1c and Stx2b are mainly associated with diarrheal disease [49,50]. In this study, the most common Stx subtype was Stx1c and this was linked to diarrheal disease. Stx1 is the most common type (50.4%) found among healthy adults in Japan, although the exact Stx1 subtypes are unclear [37]. The prevalent Stx subtype among asymptomatic humans remains largely unknown in China. Non-O157 STEC isolated from diarrheal patients in China were dominated by the *stx1c* subtype [41], which is accordant with our *stx* subtype from asymptomatic food handlers. Several studies depicted the prevalence and characteristics of STEC strains in retail raw meats in China, showing that *stx1c* and *stx2e* were predominant subtypes [51,52]. They could serve as a reservoir for the dissemination of virulence genes through the food chain. We also identified the *stx2e* subtype, which was a less common subtype in human STEC strains but was linked to edema disease in swine [53]. Some highly pathogenic *stx* compositions (i.e., *stx2c*, *stx2d*) were not present in our strains [41]. The combination of the *stx2* and *eae* genes was also not found in our strains. The *stx2* and *eae* genes together are often found to be associated with hemolytic uremic syndrome and are considered a risk factor for high virulence [54].

The Stx expression is crucial for STEC pathogenesis [55]. We found that the Stx inducibility of the ten strains was diverse. The Stx1-producing strains were non-inducible or less inducible, while all of the Stx2-producing strains were inducible. A study showed that the Stx1-encoding phage is less sensitive to inducing agents than the Stx2-encoding phage and that the level of Stx1 production is lower than that of Stx2 induced by mitomycin C [56]. The expression of Stx1 is regulated by two types of independent promoters. The first is a late phage promoter pR’ that depends on phage induction; this allows the expression and release of the Shiga toxin via the bacteriophage-mediated cell lysis. The other is a special Stx1 promoter, containing a binding site for Fur protein, which makes complexes with iron. Therefore, Fur represses Stx1 expression in the presence of iron, while Stx1 is expressed in the absence of iron. This regulation is completely independent of phage induction, and Stx1 levels of production are similar to those observed under conditions where the Stx1 phage is not induced [57]. However, a different level of Stx production can be observed for Stx2-encoding phages. The Stx2 production is always dependent on phage induction, whereas Stx release is dependent on cell lysis [58]. The strain STEC1588 (O112:H19, *stx1a*+*stx2a*) showed a higher Stx2 transcription level than other Stx2 subtype strains, partly indicating that the Stx2a subtype has the potential to cause serious disease.

The Stx phage may play a major role in the development of pathogenic STEC-mediated disease. Although Stx exhibits differences in sequence features, host specificity, and clinical outcomes, the *stx* genes are all located downstream of the late phage gene promoter. The Stx phage showed a modular construction and sequence heterogeneity, which was in accordance with our study [59]. The 14 Stx prophages showed considerable genomic diversity in terms of genome size and content, structure, and insertion site. Moreover, the prophages with the same insertion site and the same subtype of Stx, the structure and composition are also not entirely identical. It was reported that an STEC strain may carry multiple and different Stx phages [60]. We found that four strains harbored two Stx prophages. The Stx phage integrases seemed to have evolved to recognize specific sites inserted within the bacterial chromosome. Therefore, although one Stx phage integrates preferentially at one specific site, the integrase is able to recognize secondary sites for the integration of another phage genome if the preferred site is occupied or deleted [61]. We found seven different insertion sites in the 14 Stx prophages [53,59], and the *dmsB* and *yfhL* were the newly identified insertion sites in this study, indicating that more genes can act as the integration site of Stx phages.

Some studies have indicated that β-lactams, quinolones, trimethoprim, and trimethoprim-sulfamethoxazole can cause bacterial SOS reactions (DNA damage response pathway) and induce Shiga toxin production. However, other studies have suggested that certain antimicrobials, such as tetracycline, azithromycin, fosfomycin, and chloramphenicol, can block the SOS response and Stx production, such that they have been used to treat STEC infection to prevent HUS [62,63]. Among these potential antibiotic classes, resistance to tetracycline was observed in our study. However, all of the isolates were susceptible to azithromycin and chloramphenicol, which might be considered for the treatment of STEC infections when necessary. STEC strains can transfer their resistance to other strains through mobile genetic determinants [64]. Therefore, it is important to monitor AMR in STEC isolates and prevent the overuse of antibiotics. 

The whole-genome phylogeny indicated a high diversity of STEC strains isolated from asymptomatic food handlers. Only three strains clustered closely together with less than 10 SNPs distance. When compared with strains from different sources, the strains from asymptomatic food handlers clustered with strains of diarrhea patients and environment-derived strains, but they were genetically distant from the strains of HUS patients. This may suggest that strains from asymptomatic food handlers were less pathogenic and posed a lower potential to cause severe disease in humans.

To our knowledge, this is the first study reporting the prevalence and characteristics of STEC strains isolated from asymptomatic food handlers in China. MLST types, serotypes, *stx* subtype, prophages, and Stx transcription levels suggest that STEC from asymptomatic carriers in China are highly diverse. The whole-genome phylogeny indicated that the strains from asymptomatic food handlers may pose a low pathogenic potential.

## 4. Materials and Methods

### 4.1. Sample Collection and Bacterial Strains

The sampling of asymptomatic food handlers (workers in the industry of food processing and catering companies, and cookers in restaurants) for physical examinations was carried out in three different geographical regions in China. Briefly, 49 anal swabs were collected from Yushu, Qinghai Province in 2013; a total of 1349 anal swabs were collected from Shenzhen, Guangdong Province from 2014 to 2016; and 2589 anal swabs were sampled from Yulin, Guangxi Zhuang Autonomous Region in 2022. Anal swab samples were collected in 15 mL sterile tubes and transported using ice packs to the laboratory at the National Institute for Communicable Disease Control and Prevention, China CDC, for the isolation of STEC. All samples were enriched in EC broth (Land Bridge, Beijing, China), and then enrichments were plated onto MacConkey agar (Oxoid, Hampshire, UK) and CHROMagar^TM^ ECC agar (CHROMagar, Paris, France). The isolates were examined by PCR for the presence of the *stx1* and *stx2*; all *stx*-positive isolates were confirmed as *E. coli* using the API-20E system (bioMérieux, Lyon, France), as previously described [65].

### 4.2. Whole Genome Sequencing (WGS) and Assembling

Genomic DNA was extracted using the Wizard Genomic DNA purification kit (Promega, WI, USA) according to the manufacturer’s instructions. Whole-genome sequencing was performed using two platforms; the 150 bp paired-end short reads were sequenced via the MGISEQ-2000 platform (MGI Tech Co., Ltd., Shenzhen, China). The 10 kb long library was sequenced via the PacBio Sequel platform (Pacific Biosciences, Menlo Park, CA, USA). The low-quality reads (Q-value ≤ 20 for short reads or length ≤ 1000 bp for long reads) and adapter sequences were filtered. The long reads were preliminarily corrected using Canu (v1.5). The assembling was based on the corrected reads using Canu and Falcon (v0.3.0). GATK (v1.6-13) was used to correct the single base error in assembling short reads.

### 4.3. WGS-Based Molecular Characterization

In silico *stx* subtyping was conducted by comparing genome assemblies against our in-house *stx* subtyping database that included all identified *stx1* and *stx2* subtypes using ABRicate version 0.8.10 with default parameters. SerotypeFinder (https://cge.food.dtu.dk/services/SerotypeFinder/, accessed on 20 March 2023) was used to determine the serotypes. The detection of the virulence genes and antimicrobial resistance genes was performed by comparing assemblies against the *E. coli* virulence factor database (https://github.com/phac-nml/ecoli_vf, accessed on 20 March 2023) and the Comprehensive Antibiotic Resistance Database (http://arpcard.mcmaster.ca, accessed on 28 March 2023), respectively, using ABRicate with default parameters (coverage ≥ 80% and identity ≥ 80%). Multilocus sequence typing (MLST) of the isolate was conducted through an online tool provided by the Warwick *E. coli* MLST scheme (https://enterobase.warwick.ac.uk/species/ecoli/allele_st, accessed on 5 April 2023).

### 4.4. Genomic Characterization of Stx-Converting Prophages

The Stx prophage sequences were extracted from genomes and characterized using the previously described methods [66]. Briefly, the PHAge Search Tool Enhanced Release (PHASTER, http://phaster.ca/, accessed on 2 June 2023) was used to identify the Stx prophages. The genome annotation of the Stx prophages was predicted by the RAST server (http://rast.nmpdr.org/, accessed on 2 June 2023). The gene adjacent to the integrase was defined as the phage insertion site [59]. The Stx prophages were compared and visualized in detail using Easyfig [67].

### 4.5. RNA Extraction and Relative Quantification of Stx Expression

The STEC overnight cultures in Luria Bertani (LB) broth were inoculated onto fresh LB medium and incubated at 37 °C with shaking until OD_600_ was about 0.6. Each culture was subdivided into two flasks, and mitomycin C was added to one of the subcultures at a final concentration of 0.5 μg/mL. Both induced and non-induced cultures were continuously grown at 37 °C with shaking for 3 h, and then the total RNA was extracted using RNeasy Mini Kit (Qiagen, Hilden, Germany) [30]. Reverse-transcription quantitative PCR (RT-qPCR) was performed on a Rotor-Gene Q Real-Time PCR system (Qiagen, Hilden, Germany) using the One Step TB Green^®^ PrimeScript^TM^ RT-PCR kit (TaKaRa, Dalian, China) according to the manufacturer’s instructions. The primer pairs for *stx1* (5′-GGAATTTACCTTAGAYTTCTCRAC-3′ and 5′-CCTGTGCCACTATCAATCATC-3′), *stx2* (5′-TCCATGACAACGGACAGCAG-3′ and 5′-ACGCCAGATATGATGAAACCAG-3′), and for the housekeeping gene *gapA* (glyceraldehyde-3-phosphate dehydrogenase A) (5′-TATGACTGGTCCGTCTAAAGACAA-3′ and 5′-GGTTTTCTGAGTAGCGGTAGTAGC-3′) were used in the real-time PCR. The *gapA* gene was used for a within-sample normalization. The DDCt method was used to determine the relative amount of Stx mRNA expression compared to *gapA* [68]. The Stx expression levels under mitomycin C-inducing relative to non-induced ones were calculated.

### 4.6. Antimicrobial Susceptibility Test

The minimal inhibitory concentrations (MICs) were determined via the broth microdilution method using the BD Phoenix^TM^ M50 Automated Microbiology System (BD, Franklin Lakes, NJ, USA). The following 20 antimicrobial agents were tested: amikacin (AMI, 4–64 µg/mL), ampicillin (AMP, 2–32 µg/mL), ampicillin-sulbactam (AMS, 2–32 µg/mL), aztreonam (ATM, 0.25–16 µg/mL), cefotaxime (CTX, 0.25–16 µg/mL), cefoxitin (FOX, 2–64 µg/mL), ceftazidime (CAZ, 0.25–16 µg/mL), chloramphenicol (CHL, 4–32 µg/mL), ciprofloxacin (CIP, 0.015–2 µg/mL), colistin (CT, 0.25–8 µg/mL), ertapenem (ETP, 0.25–8 µg/mL), imipenem (IPM, 0.25–8 µg/mL), meropenem (MEM, 0.125–2 µg/mL), nalidixic (NAL, 4–32 µg/mL), nitrofurantoin (F, 32–256 µg/mL), tetracycline (TET, 1–16 µg/mL), Tigecycline (TIG, 0.25–8), Streptomycin (STR, 4–32), trimethoprim-sulfamethoxazole (SXT, 0.5–8 µg/mL), and ceftazidime-avibactam (CZA, 0.25/4–8/4 µg/mL). The strains were defined as being resistant, intermediate, or susceptible according to the standard reference values (Clinical Laboratory Standards Institute [CLSI], 2022).

### 4.7. Phylogenomic Analysis

A whole-genome single-nucleotide polymorphism (wgSNP) phylogeny was used to assess the genomic diversity and relatedness of 10 STEC strains obtained in this study as well as 32 strains downloaded from the National Center for Biotechnology Information (NCBI) database. The core SNPs were obtained using Snippy version 4.3.6 (https://github.com/tseemann/snippy, accessed on 5 June 2023) with default parameters. Gubbins v2.3.4 [69] was then used to remove recombination from core SNPs and construct a maximum likelihood tree based on the filtered SNP alignments. The SNPs distances were assessed using snp-dists v0.7.0 (https://github.com/tseemann/snp-dists, accessed on 5 June 2023). The visualization and annotation of the phylogenetic tree were conducted by using an online tool, ChiPlot (https://www.chiplot.online/#Phylogenetic-Tree, accessed on 6 June 2023) [70].

### 4.8. Statistical Analysis

Statistical analysis was performed using SPSS 26.0. Dunnett’s *t*-test was carried out to compare Stx2 expression levels. There were statistically significant *p*-values ≤ 0.05.

### 4.9. Data Availability

The complete genomes of ten STEC strains are available at GenBank under the accession numbers listed in Figure 3.

## Figures and Tables

**Table 1:** Prevalence of STEC in anal swab samples from food handlers in China.

Sampling Site	No. of Samples	No. of STEC Isolates	Prevalence of STEC (%)
Guangdong	1349	3	0.222
Qinghai	49	1	2.041
Guangxi	2589	6	0.232
Total	3987	10	0.251

**Table 2:** Characteristics of STEC derived from asymptomatic food handlers in this study.

Strain	Serotype	Sequence Type	*stx* Subtype	Sampling Year	Antimicrobial Resistant Phenotypes	Antimicrobial Resistant Genes
STEC434	O43:H2	937	*stx2d*	2014	-	-
STEC435	O91:H14	33	*stx1a+stx2b*	2013	-	-
STEC438	ONT:H21	40	*stx1c*	2014	Streptomycin, Tetracycline	*ant(3*″*)-Ia*, *tet(A)*
STEC509	O8:H19	21	*stx2e*	2016	Tetracycline	*tet(B)*
STEC1585	O178:H7	278	*stx1c*	2022	-	*-*
STEC1586	O112ab:H8	75	*stx1c+stx2b*	2022	-	*fosA7*
STEC1587	O21:H25	971	*stx1a*	2022	-	*-*
STEC1588	O112ac:H19	2038	*stx1a+stx2a*	2022	-	*-*
STEC1589	O112ab:H8	75	*stx1c*	2022	-	*fosA7*
STEC1590	O112ab:H8	75	*stx1c+stx2b*	2022	-	*fosA7*

‘-’, susceptible (antimicrobial resistant phenotypes) or absent (antimicrobial resistant genes).

## Data Availability

The data presented in this study are available by contacting the corresponding author upon reasonable request.

## Supplementary Materials

The following supporting information can be downloaded at: https://www.mdpi.com/article/10.3390/toxins15110640/s1, Table S1: Characterization of 14 Stx prophages in this study.
