# Supplementary material for: Characteristics of Shiga Toxin-Producing Escherichia coli Circulating in Asymptomatic Food Handlers
Source: Toxins (Basel). 2023 Nov 2;15(11):640. doi: 10.3390/toxins15110640 (PMC10675304; doi:10.3390/toxins15110640)
Supplement: Supplementary file 1 [file toxins-15-00640-s001.zip › toxins-2662704-supplementary.pdf]

Table S1. Characterization of 14 Stx prophages in this study.

Strain	Stx subtype	Lenth(bp)	GC content	No. of CDS	tRNA
STEC434	*stx2d*	49648	52.1	72	3
STEC435	*stx1a*	48828	49.6	71	0
STEC435	*stx2b*	46213	52.7	61	1
STEC438	*stx1c*	64251	49.7	79	0
STEC509	*stx2e*	55219	48.9	89	3
STEC1585	*stx1c*	62413	49.7	77	0
STEC1586	*stx1c*	62816	49.6	85	0
STEC1586	*stx2b*	46072	53.4	64	1
STEC1587	*stx1a*	45826	52.3	65	0
STEC1588	*stx1a*	48278	50.9	72	0
STEC1588	*stx2a*	45120	52.5	63	3
STEC1589	*stx1c*	63085	49.6	82	0
STEC1590	*stx1c*	62821	49.6	81	0
STEC1590	*stx2b*	47889	53.1	65	1

“-”, The insertion site is undetermined

Insertion site
yccA
yecE
yfhL
dmsB
yecE
wrbA
dmsB
yfhL
potC
-
dusA
dmsB
dmsB
yfhL
